# Pseudo‐Haptic Feedback: Multi‐Modal Approaches to Stiffness Perception and User Experience

**DOI:** 10.1002/advs.78306

**Published:** 2026-10-20

**Authors:** Mehmet Ege Cansev, Xiaodong Sun, Gionata Salvietti, Domenico Prattichizzo, Philipp Beckerle

**Affiliations:** ^1^ Chair of Autonomous Systems and Mechatronics Department of Electrical Engineering Friedrich‐Alexander‐Universität Erlangen‐Nürnberg Erlangen Germany; ^2^ Department of Electrical Engineering Friedrich‐Alexander‐Universität Erlangen‐Nürnberg Erlangen Germany; ^3^ Department of Information Engineering and Mathematics University of Siena Siena Italy; ^4^ Department of Artificial Intelligence in Biomedical Engineering Friedrich‐Alexander‐Universität Erlangen‐Nürnberg Erlangen Germany

**Keywords:** force feedback, pseudo‐haptic feedback, stiffness perception, user experience, virtual reality

## Abstract

Pseudo‐haptics is a technique that creates the illusion of haptic sensations in virtual environments by leveraging visual feedback and the interplay between visual and haptic perception. Pseudo‐haptic feedback can enhance virtual reality interactions by providing perceptual cues that substitute for or complement physical haptic feedback. This work extends a 3D deformation rendering approach to generate visual deformation cues for stiffness perception in virtual reality and evaluates their impact on user experience through a user study measuring presence and workload. Results from a user study (n=20) indicate that deformation cues support stiffness perception at levels comparable to physical haptic feedback in both objective and subjective measures. Combining pseudo‐haptic and force feedback further enhances stiffness perception accuracy and presence without increasing workload. These findings demonstrate that well‐designed pseudo‐haptic feedback mechanisms can effectively complement or substitute for force feedback in virtual reality interactions.

## Graphical abstract

Can visual deformation substitute for physical haptic feedback? In immersive VR, deformation‐based pseudo‐haptics enables stiffness discrimination comparable to wearable force feedback, while their combination yields the highest performance and presence without increasing workload. The results highlight the potential of multi‐modal feedback for efficient stiffness rendering.

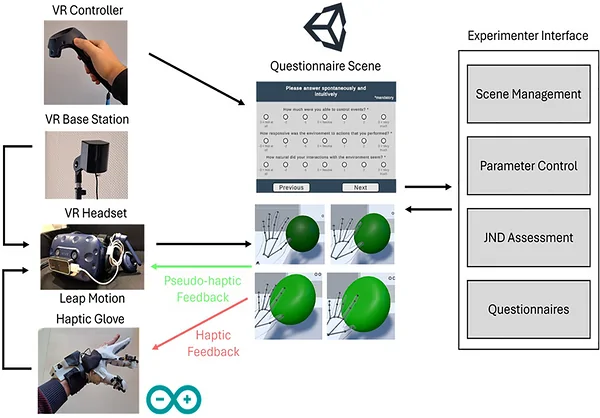

## 1 Introduction

Communication technologies have greatly improved the transfer of visual and auditory information over distances as demand has increased [1]. In contrast, realistic communication of haptic information remains challenging, motivating the development of wearable and virtual haptic interfaces [2, 3, 4].

A wide range of haptic devices has been developed for applications ranging from medical systems to driving assistance [5, 6]. While grounded devices can provide high‐quality feedback, their limited portability restricts their use in everyday scenarios [7]. Consequently, wearable haptic interfaces, particularly for the hands, have attracted considerable attention as a means of delivering haptic information during natural interaction. However, wearable solutions involve inherent trade‐offs between device size, comfort, cost, and rendering fidelity [8]. The need for lightweight actuators and compact form factors can limit feedback quality, while bulky components and limited adaptability to different users may reduce comfort and usability [7, 9]. These challenges motivate the exploration of alternative approaches for conveying haptic information with reduced hardware requirements.

Most of these issues could be resolved by delivering haptic information without requiring any mechanical haptic interface, which can be achieved by pseudo‐haptics. Lécuyer [10] defined pseudo‐haptics as a technique that creates the illusion of haptic sensations in virtual environments by leveraging visual feedback and the interplay between visual and haptic perception. By intentionally altering visual cues, it influences and distorts users' tactile perception, effectively simulating haptic feedback through visual means and utilizing the phenomenon of haptic illusions [10].

Various visual stimuli have been used to reflect haptic properties such as, weight, force, compliance, roughness, and friction [9]. Later on, the concept has been extended to include other sensory channels such as auditory feedback [11]. Recent advancements in virtual reality (VR) and augmented reality (AR) technologies have set the ground for a more realistic and immersive experience in many application domains [12]. Recent work has highlighted the importance of user‐centered design in intelligent systems, where both algorithmic performance and user experience must be jointly optimized [13]. This perspective is particularly relevant in pseudo‐haptic systems, where perceptual realism depends heavily on human sensory integration. With the help of VR, pseudo‐haptic feedback methods can enhance the experienced sense of presence, i.e., the subjective experience of being in the virtual environment rather than in the physical location where the user actually resides [14, 15].

In this work, we focus on vision‐based pseudo‐haptic feedback techniques to simulate the stiffness of objects in a virtual environment. We introduce a virtual deformation algorithm assisted by color changes of the deformed virtual object in VR to convey haptic information through visual pseudo‐haptic cues. To enhance the relatively low‐fidelity feedback of wearable haptic devices, we propose a multi‐modal feedback approach that integrates visual pseudo‐haptics and physical haptic feedback. In this context, the term multi‐modal refers to the integration of visual pseudo‐haptic cues and physical haptic force feedback, rather than the combination of multiple pseudo‐haptic sensory channels, e.g., visual and auditory cues. The approach is evaluated against traditional force‐feedback methods by comparing stiffness perception accuracy and user experience across different feedback modalities, including haptic feedback, visual pseudo‐haptics, and their combinations. This work contributes a deformation‐based pseudo‐haptic rendering approach for stiffness perception in VR and, more importantly, a systematic evaluation of how visual pseudo‐haptic and wearable force‐feedback cues interact to influence stiffness discrimination, presence, and workload. The findings provide practical insights for the design of multi‐modal interfaces in immersive VR.

## 2 Related Works

Pseudo‐haptic applications can engage multiple sensory channels to create the illusion of haptic sensations. Consequently, it is essential to understand how multisensory information is processed by the human perceptual system, as pseudo‐haptic feedback relies on the integration of visual, proprioceptive, and tactile cues to shape perception and user experience.

### 2.1 Pseudo‐Haptics

Pseudo‐haptic feedback aims to convey haptic information in the absence of direct tactile stimulation primarily through visual cues. The visual representation of the interactive environment provides haptic cues that we relate to the physical laws as perceptual effects or metaphors [16, 17]. In many scenarios, the visual information is integrated with haptic feedback, as well as with proprioception, the sensation of body position and self‐movement [18]. In the latter case, pseudo‐haptic feedback is generated through the contradiction between the “observed” and “expected”, frequently referred to as the control‐display (C/D) ratio [17, 19, 20, 21]. Dominjon et al. [19] showed that manipulating the C/D ratio of a virtual ball on the display can alter the perceived weight of a real ball. In their study, participants lifted the real ball without visual feedback while observing the motion of its virtual counterpart. They even showed that a heavier ball can be perceived as lighter than an actually lighter ball. To manipulate the C/D ratio and the perception of material properties, a physical object is not always required. For example, the Touchy interface uses a co‐localized cursor on a touch screen that can be moved and/or visually deformed to evoke haptic properties, such as, friction, hardness, and roughness [22]. This interface illustrates the inexpensive and simple nature of pseudo‐haptics with potential to deliver variety of haptic information.

Pseudo‐haptic feedback can also be complementary to tactile feedback through multisensory integration, in which information from different sensory systems is combined to shape perception and decision making [23, 24]. When the visual cues are presented simultaneously with proprioception and/or haptic feedback (cutaneous or kinesthetic), the resulting multisensory integration can reinforce the illusion of physical interaction, leading users to perceive variations in different physical properties of objects such as weight [25, 26] and stiffness [27, 28]. Stellmacher et al. [26] tested the effect of different feedback modalities, i.e., only visual pseudo‐haptic feedback with its combination with vibration feedback, on weight perception as well as user experience of the interaction. Compared to baseline condition, where no weight simulation was shown, both feedback modalities improved the sense of weight change and user experience, while the vibrotactile feedback from the VR controller did not further improve the weight perception compared to pseudo‐haptic feedback. Similarly, Hirao et al. [29] demonstrated that vibration applied to the wrist and elbow tendons enhances pseudo‐haptic perception by extending the tolerance for visual–physical discrepancies, effectively increasing the illusion's robustness. An alternative approach for weight perception was presented using electrical muscle stimulation [25]. The results showed that combining the C/D ratio manipulation with somatosensory stimulation improves the weight perception significantly.

Alternatively, pseudo‐haptic feedback can also be achieved through mid‐air interactions with virtual objects using free‐hand gestures, without providing any physical haptic cues to users. While mid‐air haptic technologies, such as ultrasound‐based feedback, have also been developed to provide tactile sensations without physical contact [30], pseudo‐haptic techniques instead rely on visual cues and interaction dynamics to evoke haptic sensations. The advancements in VR systems provide a suitable ground for touchless, gesture‐based kinesthetic interactions [31]. Free‐hand tracking techniques combined with precisely controlled visual distortions recently extend the traditional pseudo‐haptic approaches. Kim and Xiong [32] proposed four different pseudo‐haptic features in a VR environment to simulate a button push action where the participants were moving their hands freely in space. Their results showed that all proposed features positively affected the user experience of the haptic illusion, improving perceived realism, interaction quality, and user satisfaction compared to a condition without pseudo‐haptic feedback. They further extended their work to implement a virtual keyboard with pseudo‐haptic features which similarly improved the user experience of typing except the perceived workload [33]. Not only visual cues, but auditory cues were also used to create pseudo‐haptic effect during mid‐air interactions [34, 35]. Gupta et al. [36] compared mid‐air text input with audio‐visual pseudo‐haptic feedback and with vibrotactile feedback delivered through wrist‐ or ring‐worn wearable devices, and found that while performance metrics remained unchanged, participants strongly preferred the tactile condition, which was reflected in lower mental demand and effort ratings of NASA Task Load Index (NASA‐TLX) [37]. Similarly, earlier work by Weber et al. [38] compared force, visual, and vibrotactile feedback during collision tasks in VR. Force feedback yielded higher task performance than both visual and vibrotactile feedback, while the latter two showed comparable performance. They also reported that visual feedback led to higher perceived workload compared to haptic feedback modalities.

### 2.2 Pseudo‐Haptics in Stiffness Perception

Beyond weight and friction perception, recent studies have applied pseudo‐haptic techniques to modulate the perception of stiffness. By altering the visual deformation of objects or the control–display (C/D) ratio during interaction, users can perceive variations in stiffness even in the absence of real physical forces. For instance, Weiss et al. [39] demonstrated that manipulating the C/D ratio of a hand's movement when pressing virtual objects could induce perceived changes of up to ±30% in stiffness, without the participants detecting any inconsistency. Similarly, Bouzbib et al. [40] quantified the perceptual thresholds of pseudo‐stiffness by visually deforming the virtual representation of a rigid tangible object in VR while the user interacted with a non‐compressible physical object. Their results showed that visual‐only cues can effectively induce the perception of different stiffness levels, with the perceived range depending strongly on the scale of the interaction and applied force. While they demonstrated pseudo‐stiffness effects through visually induced deformation of a rigid tangible object, the present work investigates stiffness perception through localized deformation of a fully virtual object during mid‐air interaction. Furthermore, we examine how such deformation interacts with wearable force feedback in immersive VR.

Recent multisensory approaches demonstrate that combining pseudo‐haptic cues with complementary tactile or proprioceptive feedback enhances stiffness perception in virtual environments. The Holitouch system [27] integrated visual displacement cues with fingertip vibrations and muscle‐induced joint resistance to reproduce button stiffness without grounded actuators. Pezent et al. [28] similarly coupled visual feedback with wrist‐squeeze actuation, using C/D ratio manipulation to render mid‐air stiffness variations. Extending this principle, Basdogan et al. [41] showed that altering the visual–haptic relationship in a cable‐driven interface could expand the perceived stiffness range beyond the device's physical limits. Likewise, Achibet et al. [42] combined passive fingertip constraints with pseudo‐haptic motion scaling in the FlexiFingers system, enabling multi‐finger stiffness perception through simple C/D ratio adjustments. These approaches induce stiffness perceptions by altering the user's movement trajectory. In contrast, our approach modifies the geometry of the virtual object itself through localized mesh deformation coupled to the interaction physics. Such object‐centered deformation may enhance perceptual fidelity by providing spatially and temporally coherent visual cues at the point of contact, as the visual response remains directly tied to the contact region rather than to global motion remapping.

Deformation‐based pseudo‐haptics has been widely explored as an intuitive way to convey material stiffness in virtual and augmented environments. Early work by Argelaguet et al. [43] introduced the concept of elastic images, in which visual deformation of 2D elements created a convincing illusion of local elasticity without any physical feedback. Extending this approach, Punpongsanon et al. [44] developed SoftAR, a spatial augmented reality system that visually manipulated both object deformation and surface color to alter perceived softness, showing that combined geometric and photometric cues can bias perceived material properties. Achibet et al. [45] also used color as visual feedback in virtual grasping scenarios. Similar principles were adapted for mobile and handheld devices, where visual deformation of virtual or on‐screen objects was coupled with user input through touch or grasping motions [46]. These studies consistently demonstrated that reducing the amount of visual deformation relative to the applied motion increased perceived stiffness, while exaggerated deformation led to softer impressions. Kawabe [47] demonstrated that simply observing the elongation of a virtual object during mid‐air actions could generate strong stiffness illusions without any tactile device. In an alternative approach, Ban et al. [48] modulated the visual deformation of the user's virtual hand to manipulate perceived stiffness when pinching virtual objects. Together, these studies highlight that visual deformation provides a lightweight alternative to physical haptic feedback for both individual and shared VR interactions. Still, prior studies primarily manipulated perceived compliance through visual appearance changes, hand deformation, or altered visual–haptic relationships [41, 44, 48], the present work focuses on localized object mesh deformation as a cue for stiffness perception in immersive VR.

More recently, Kasai et al. [49] employed physics‐based mesh deformation to provide visual feedback during grasping manipulation, demonstrating the potential of localized geometric deformation for object interaction and contact awareness. In contrast, the present work investigates mesh deformation as a pseudo‐haptic cue for communicating material stiffness and combines it with a dynamically updated interaction geometry and wearable force feedback. Existing pseudo‐haptic approaches for stiffness perception predominantly induce stiffness illusions through image‐based deformation or motion‐remapping techniques, while comparatively fewer studies have investigated localized mesh deformation of the object itself as a cue for material stiffness. Furthermore, limited attention has been given to maintaining consistency between the visually deformed object and the interaction geometry. In addition, relatively few studies investigate how such visual cues interact with wearable haptic feedback in immersive VR settings. This motivates exploring hybrid feedback strategies that combine mesh‐based visual deformation cues with wearable force feedback to enhance stiffness perception and user experience.

### 2.3 Research Focus

In summary, prior work has established the potential of visual pseudo‐haptics and hybrid feedback for stiffness simulation. However, most studies have focused either solely on visual illusions or on physical haptic devices, without systematically comparing different feedback modalities under identical task conditions. Moreover, systematic investigations of fingertip‐level stiffness discrimination that integrate both visual pseudo‐haptic cues and wearable force feedback in immersive VR remain largely unexplored.

To address these gaps, we introduce an immersive VR‐based experimental framework that combines deformation and color‐based pseudo‐haptic feedback with fingertip force feedback delivered through a wearable glove. By evaluating task success rate in stiffness discrimination as an objective performance metric, together with subjective measures of presence and workload across five feedback modalities, this study aims to systematically characterize how pseudo‐haptic and physical feedback interact to shape stiffness perception and user experience in immersive VR. Based on these considerations, we formulate the following hypotheses:

H1.1
Task success rates under pseudo‐haptic feedback conditions are comparable to those achieved with haptic feedback alone.
H1.2
Task success rates increase as additional pseudo‐haptic modalities are combined with haptic feedback.
H2.1
Presence ratings under pseudo‐haptic feedback conditions are comparable to those observed with haptic feedback.
H2.2
Presence ratings increase as additional pseudo‐haptic feedback modalities are presented alongside haptic feedback.
H3
Incorporating pseudo‐haptic feedback modalities increases workload, particularly perceived mental demand and effort, compared to haptic feedback alone.

**H3** was motivated by inconsistent findings in the literature. While some studies reported increased mental demand and effort associated with visual pseudo‐haptic feedback compared to tactile or force feedback modalities [36, 38], others found no significant workload differences across feedback conditions [26, 33]. Therefore, H3 was included to examine whether incorporating pseudo‐haptic cues alongside force feedback influences perceived workload in the present stiffness discrimination task.

## 3 Methodology

The proposed system enables interaction with deformable virtual objects in VR using a combination of visual pseudo‐haptic cues and wearable force feedback. Deformation‐based and color‐based visual cues are integrated with fingertip force feedback delivered through a haptic glove. A controlled user study evaluates the effectiveness of these feedback modalities for stiffness perception and user experience.

### 3.1 System Overview

The system, illustrated in Figure 1, combines a HTC Vive Pro headset (HTC, Taoyuan, Taiwan), a Leap Motion hand tracking device (Leap Motion Inc., San Francisco, CA, United States), the Unity engine (Unity Technologies, San Francisco, CA, United States), and a custom haptic glove controlled by Arduino Uno (Arduino, Ivrea, Italy) to create immersive virtual interactions. The Vive Pro provides high‐resolution visuals with precise positional tracking, while the Leap Motion captures real‐time hand movements. The haptic glove delivers force feedback through servo‐driven actuators, responding to signals from the virtual environment. In Unity, deformable spheres were simulated with varying stiffness and visual cues for pseudo‐haptic feedback. Questionnaires regarding user experience of participants were also integrated into VR in the user study.

**FIGURE 1:**
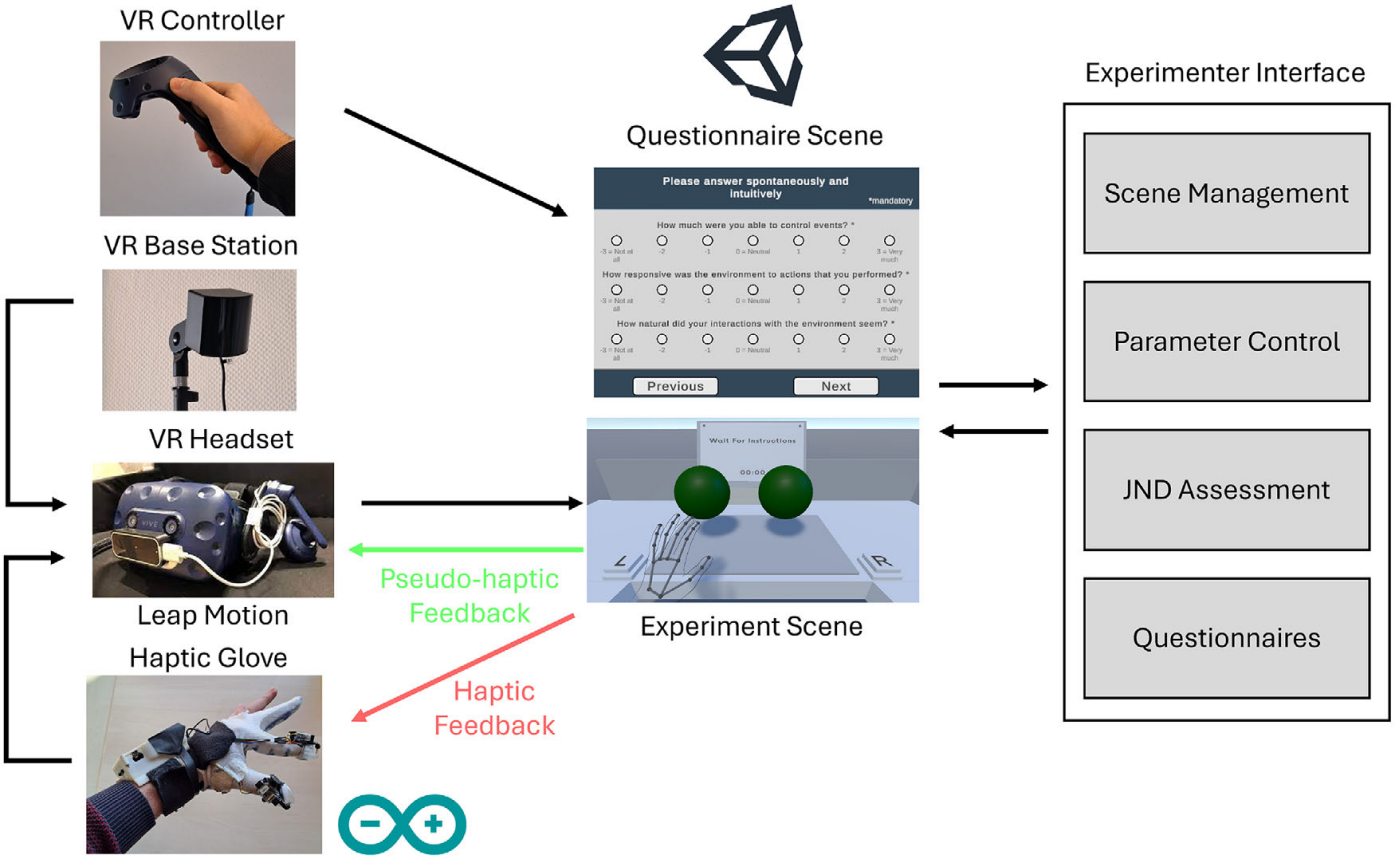
System overview of the VR interaction framework. The user interacts with virtual objects through the VR headset and Leap Motion hand tracking while wearing a haptic glove that provides fingertip force feedback. Visual pseudo‐haptic cues (deformation and color change) and haptic feedback are generated in the Unity‐based virtual environment. The experimenter controls the experiment through a separate interface that manages scene parameters, JND assessment, and questionnaires.

### 3.2 Hand Tracking in Virtual Reality

Achieving real‐time, realistic hand tracking is a prerequisite to the natural interaction with the environment. To achieve this, the Leap Motion device is mounted at the center of the HTC Vive Pro headset. The device employs stereo infrared cameras to estimate 3D hand and finger joint positions using computer vision algorithms. This mounting method ensures accurate hand tracking by positioning the sensor to cover the hand interaction area without obstructing tracking volume for the Leap Motion sensor. Using the Ultraleap Hand Tracking Software and Ultraleap Unity Plugin, hand tracking is enabled, allowing real‐time generation of tracked hand models within the virtual environment. We utilized the Ultraleap‐developed Physical Hand Model, which precisely represents the motion of the entire hand using 26 data points and includes interpenetration blocking functionality, which prevents the virtual hand from passing through rigid bodies in the VR environment. Additionally, compared to realistic human hand models, this model should effectively mitigate uncanny valley effects [50].

### 3.3 Pseudo‐Haptic Feedback Through Deformation

During the development of the sphere deformation algorithm, we referred to the mesh deformation tutorial by Flick [51] and modified the implementation to simulate soft spheres with varying stiffness. By integrating stiffness parameters with color changes and continuous force feedback, users can intuitively perceive sphere stiffness in the virtual environment, enhancing realism. We also developed a dynamic collider radius adjustment mechanism that updates the collider size based on the current mesh deformation, allowing the fingertip to follow the indentation during interaction. In addition, a moving average filter was applied to reduce deformation instability caused by minor hand tremors, improving interaction stability.

In Unity, the mesh of a sphere defines its shape and appearance. It includes data, such as vertices, triangles, normal vectors, and their coordinates, which determine how the sphere is rendered. Visual geometry and physical interaction are handled separately. While the mesh defines the rendered shape of the object, collisions are detected using a collider that approximates the object's physical boundary. To simulate deformation, such as a sphere being pressed by a finger, the positions of neighboring vertices of the contact point in the mesh are modified. This requires real‐time calculation and updating of the vertex positions using deformation algorithms. Figure 2 demonstrates the effect of deformation algorithm on different vertices.

**FIGURE 2:**
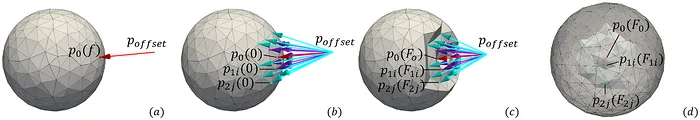
(a) The point of contact with the sphere is denoted as $p_{0}\left(f\right)$ while $p_{\text{offset}}$ represents a fixed offset position along the normal at $p_{0}\left(f\right)$. The red arrow illustrates the vector $p_{0}\left(f\right)-p_{\text{offset}}$, which defines the direction of the contact force f. Mesh elements are exaggerated for clarity. (b) For a clear demonstration, the vertices are defined as neighbors with two indices. The first neighbors of $p_{0}$, denoted as $p_{1i}$, and the second neighbors, $p_{2j}$, are shown with their respective distance vectors in purple and cyan. Only two layers of neighbors are illustrated for simplicity. (c) As forces are applied to the sphere, the vertices move inward, with displacement inversely proportional to their distance from the contact point. The highest displacement occurs at $p_{0}\left(F_{0}\right)$, the contact point, while displacement decreases radially outward as a result of decreasing contact forces, i.e., $F_{2j}<F_{1i}<F_{0}$, then, $p_{2j}\left(F_{2j}\right)<p_{1i}\left(F_{1i}\right)<p_{0}\left(F_{0}\right)$. Deformations are exaggerated for visualization purposes. (d) A back view of the sphere, shown with partial transparency, highlights the deformation pattern.

Let $P=\{p_{0},p_{1},p_{2},\text{…},p_{n}\}$ be the set of vertices on a sphere of radius r, where each $p_{i}\left(0\right)=\left(x_{i},y_{i},z_{i}\right)$ lies on the sphere surface, i.e., satisfies the sphere equation $x_{i}^{2}+y_{i}^{2}+z_{i}^{2}=r^{2}$, when there are no forces applied. The point $p_{0}$ is the contact point where the maximum input force $F_{c_{0}}$ is applied.

To model the local deformation, an offset point $p_{\text{offset}}$ is introduced outside the sphere so that the contact force can be defined normal to the contact point via:

(1) $$n=\frac{p_{\text{offset}}-p_{0}}{∥p_{\text{offset}}-p_{0}∥}$$

where n is the normal vector at $p_{0}$.

Then, the force applied to a vertex, $p_{i}$, is defined as:

(2) $${F_{c}}_{i}=\frac{F_{c_{0}}}{1+∥p_{i}-p_{\mathrm{offset}}{∥}^{2}}\cdot \frac{p_{i}-p_{\mathrm{offset}}}{∥p_{i}-p_{\mathrm{offset}}∥}$$

where the second term represents a unit direction vector from the offset point toward the vertex. Distances are expressed in normalized Unity world units, making the attenuation term dimensionless. With Equation (2), $F_{c}$ radially decreases for vertices that are further away from the contact point with the direction from the offset point, $p_{\text{offset}}$, to the vertex, $p_{i}$.

For a more realistic interaction with the object, spring and damping forces, i.e., $F_{s}$ and $F_{d}$, are introduced, both acting against the contact force, $F_{c}$. Hence, the net force, $F_{n}$ acting on a vertex is

(3) $${F_{n}}_{i}={F_{c}}_{i}-{F_{s}}_{i}-{F_{d}}_{i}$$

The spring force, impeding deformation, is defined by Hooke's Law:

(4) $${F_{s}}_{i}=k\cdot \left(p_{i}\left(t\right)-p_{i}\left(0\right)\right)$$

where k is the spring coefficient. The deformation of a vertex is represented by its displacement from the undeformed position $\left(p_{i}\left(t\right)-p_{i}\left(0\right)\right)$.

Viscous damping is introduced to model energy dissipation:

(5) $${F_{d}}_{i}=c\cdot v_{i}\left(t\right)$$

where c is the damping coefficient and $v_{i}\left(t\right)$ is the velocity of the vertex. The vertex dynamics follow Newton's second law:

(6) $$m_{i}\frac{dv_{i}}{dt}={F_{n}}_{i}$$

where $m_{i}$ represent the mass associated with the vertex $p_{i}$. Assuming unit mass for each vertex and discretizing the dynamics using a forward Euler integration scheme with time step Δt, the velocity of each vertex can be calculated as:

(7) $$v_{i}\left(t+\Delta t\right)=v_{i}\left(t\right)+\left({F_{c}}_{i}-{F_{s}}_{i}-{F_{d}}_{i}\right)\Delta t$$

After substituting Equations (2), (4), (5), we can update the position of vertex as

(8) $$p_{i}\left(t+\Delta t\right)=p_{i}\left(t\right)+v_{i}\left(t\right)\Delta t$$

To create the offset from the contact point, a ray is emitted perpendicularly from the pad of the left index finger in Unity, with a length of 0.02 Unity world units. This ray detects collisions with the sphere's collider, applying a contact force $F_{c}$ at the collision point. Forces are distributed to the surrounding vertices based on Equation (2). Then, iteratively applying Equations (1)–(8) simulates the deformation and recovery of the sphere as shown in Algorithm 1.

ALGORITHM 1 **Sphere Deformation Algorithm**

**Table:** 

**Input**: vertex positions $p_{i}$, stiffness k, damping c, contact force $F_{c_{0}}$	
**Output**: updated vertex positions $p_{i}\left(t+\Delta t\right)$	
1:	**if** ray intersects with sphere collider **then** ▹ Deformation occurs
2:	
3:	**for** each vertex $p_{i}$ on mesh filter **do**
4:	Compute contact force ${F_{c}}_{i}$
5:	Compute net force ${F_{n}}_{i}$
6:	Update velocity $v_{i}\left(t+\Delta t\right)$
7:	Update position $p_{i}\left(t+\Delta t\right)$
8:	**end for**
9:	**else if** ${F_{s}}_{i}\neq 0$ **then** ▹ Restore deformation
10:	**for** each vertex $p_{i}$ on mesh filter **do**
11:	Compute net force ${F_{n}}_{i}$
12:	Update velocity $v_{i}\left(t+\Delta t\right)$
13:	Update $p_{i}\left(t+\Delta t\right)$
14:	**end for**
15:	**end if**

Based on the effect of Algorithm 1, the sphere undergoes visual deformation when pressed by a finger. However, as the depth of pressure increases, the fingertip collides with the undeformed sphere collider and does not follow the indentation into the sphere. Since the sphere is treated as a rigid body in Unity, the deformation is applied only to the visual mesh while the sphere collider remains static. As a result, the finger cannot press into the sphere as it would in reality, leading to unrealistic interaction.

To address this limitation, a dynamic sphere collider adjustment method is introduced, in which the collider radius is updated in real time to reflect mesh deformations. Since the fingertip contact point is not predetermined and deformation propagates to neighboring vertices, the vertex with the maximum displacement relative to its original position is identified at each time step. This vertex corresponds to the deepest indentation of the sphere. The distance between this point and the sphere's center, scaled by Unity's world‐space scale factor, defines the target collider radius. By continuously updating the collider in this manner, the fingertip naturally follows the indentation, enhancing realism and preventing interpenetration.

Despite its effectiveness, this approach can lead to instability. Minor hand tremors or unintentional movements cause the detected deepest point to shift frequently, especially when the sphere reaches maximum deformation. These fluctuations lead to jitter in the collider radius, which can disrupt the interaction. To improve stability, a moving average filter is applied to the target radius. By averaging the most recent values over a short window, the filter smooths out rapid fluctuations while preserving responsiveness. After iterative testing, a window size of five frames was set to achieve a balance between stability and dynamic behavior. This ensures that the collider adapts smoothly to deformations while maintaining natural interaction dynamics.

For simplicity and ease of use in the user study, the degree of deformation is represented by the relative change in the sphere's collider radius. Although the mesh deformation is applied locally around the contact point, the maximum indentation depth is approximated through a change in the collider radius, which serves as a scalar measure of deformation as a percentage:

(9) $$D\left(t\right)=\frac{r_{u}-r\left(t\right)}{r_{u}}\times 100\%$$

where $r_{u}$ and r(t) represent undeformed and filtered collider radii, respectively. We determined the operation range of 20% to 50% in pilot studies to achieve a realistic looking interaction. Depending on the virtual stiffness of the object, maximum deformation, $D_{m}$, is set within this range.

### 3.4 Pseudo‐Haptic Feedback Through Color Change

Alongside deformation, color change is a visual cue to reflect physical alterations on objects. A common example can be observed when squeezing soft objects, such as stress balls. Although the material itself does not change color, the deformation modifies how light interacts with the surface, creating subtle brightness variations that the viewer interprets as a form of visual feedback. This illustrates how even apparent color changes, caused by lighting and geometry, can enhance the perception of force and compliance. For deformable virtual objects, deliberately controlling color variation can replicate this effect more explicitly and effectively indicate the degree of deformation.

Building upon this idea, the spheres in the scene are initially rendered in green, and their colors are manipulated by adjusting its RGB values during deformation. The green component G is linearly interpolated to the collider radius r(t), defined as:

(10) G(t)=−320×r(t)+255

In the undeformed state, where r=0.5 and G=95, corresponds to a dark green color while at maximum deformation, r=0 and G=255, yields light green. Figure 3 illustrates a deformed object with color change. To implement this in Unity, the green value, G, is normalized to the range [0, 1] to match the floating‐point color representation used by the rendering system.

**FIGURE 3:**
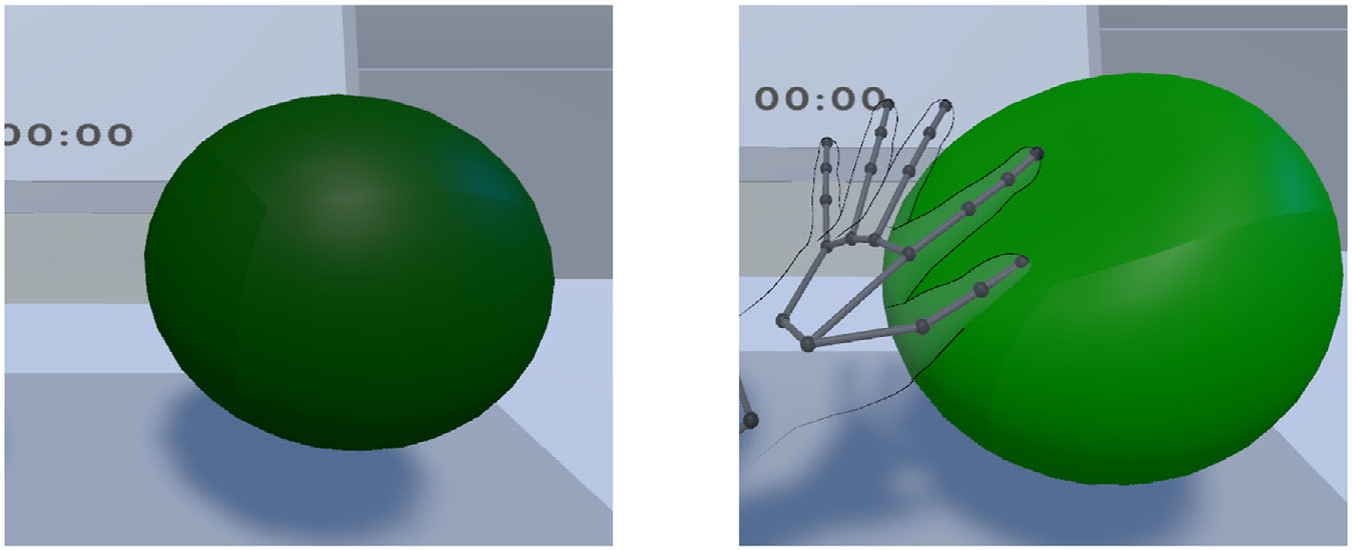
Example of pseudo‐haptic feedback through both deformation and color change.

### 3.5 Force Feedback Mechanism

To test the effectiveness of pseudo‐haptic feedback, we utilized the wearable fingertip force‐feedback device, commonly referred to as haptic thimbles, used in our previous studies [52, 53]. The device consists of two pulley systems actuated by servo motors that apply force feedback to the thumb and index fingertip. As the servo motor pulls the strings, it decreases the distance between two platforms that press against the finger, thereby increasing the force applied to the fingertip.

To align the haptic feedback with pseudo‐haptic feedback, a mapping between the smoothed collider radius r(t) and the force applied on the fingertip is needed. Therefore, we first empirically determined the applied force range on the fingertip within the operation limits of the servo motor by using a force‐sensing resistor in a voltage divider configuration. Based on this calibration, the rotation of the servo motor, θ(t), is linearly mapped to the collider radius, r(t), as follows:

(11) $$\theta \left(t\right)=\left(\theta_{u}-\theta_{m}\right)\times \frac{r\left(t\right)-r_{m}}{r_{u}-r_{m}}+\theta_{m}$$

where $r_{u}$ and $\theta_{u}$ represent the collider radius and motor rotation at the undeformed state while $r_{m}$ and $\theta_{m}$ represent the collider radius and motor rotation at the maximum deformation state of the object. $\theta_{m}$ is determined for different stiffness levels of the object based on $r_{m}$. Still, the amount of force applied with different stiffness values are optimized proportionally after pilot study evaluations.

### 3.6 Experimental Design

The study investigates how haptic and pseudo‐haptic feedback, as well as their combinations, influence stiffness perception and user experience during interaction with a virtual object. In particular, the study was designed to evaluate the hypotheses regarding differences in stiffness discrimination accuracy, experienced presence, and perceived workload across feedback modalities. The participants were presented a scene with two visually identical spheres in the virtual environment that differed in their virtual stiffness. They were asked to interact with the spheres and determine the stiffer one while experiencing different feedback modalities. Therefore, we selected a 5×1 repeated measures design where the independent variable is the feedback modality. The five conditions were deformation feedback (D), deformation combined with color change (DC), force feedback (F), deformation with force feedback (DF), and combination of deformation, color change, and force feedback (DCF). The first two conditions represent purely pseudo‐haptic feedback, the third represents purely haptic feedback, and the remaining two represent hybrid combinations.

A color‐only condition (C) and a color–force condition (CF) were deliberately excluded, as color change alone does not convey mechanical deformation and therefore provides limited information about stiffness. Since the color cue in our implementation is derived from deformation, combining color with force feedback without deformation would also be less meaningful for stiffness perception. As a result, deformation‐based conditions were prioritized when evaluating pseudo‐haptic contributions. Additionally, limiting the design to five conditions helped keep the experiment duration manageable while still enabling a systematic comparison of purely pseudo‐haptic, purely haptic, and hybrid feedback modalities.

Out of 20 trials per condition, task success rate, defined as the percentage of correct selections, was used as the objective measure of stiffness discrimination performance. Furthermore, these conditions are used to evaluate the presence of participants in the virtual environment via presence questionnaire by Witmer and Singer [14] and workload via NASA‐TLX questionnaire [37]. We used 29 questions of the presence questionnaire as is with 7‐point Likert scales, excluding three questions targeting auditory subscales, as no auditory stimuli were present in the experiment.

The study consisted of two stages. First, an individual Just Noticeable Difference (JND) calibration was performed to determine the participant‐specific stiffness discrimination threshold. This was followed by the main experiment, in which participants completed the stiffness discrimination task under the five feedback modalities and completed the questionnaires after each task. JND describes the smallest difference a person can perceive between two stimuli that differ in a single property [54]. A staircase approach was used to determine the JND for stiffness perception. In line with the practical recommendations [55], we applied one‐down/two‐up staircase method. Again, participants interacted with spheres to decide which sphere was the harder one. The maximum deformation of one sphere was always fixed to 20%, i.e., the hardest. The start value for the maximum deformation of other sphere was set to 50%, i.e., the softest, which corresponds to the largest stiffness difference at the start of the procedure, i.e., Δ=30%. The position of the harder object, i.e., the sphere on the left or right, was randomized. When the guess by the participant was correct, Δ was decreased by 5% while it was increased 10% when the guess is wrong and the Δ value is recorded as a reversal. When both objects reached the same stiffness level, i.e., when maximum deformation was set to 20%, the guess was considered wrong regardless of the selection and the trial was recorded as a reversal. The procedure was completed either when seven reversals were recorded or when the number of steps reached 40. The JND value was determined by calculating the median of the last nine Δ values. An example run is shown in Figure 4.

**FIGURE 4:**
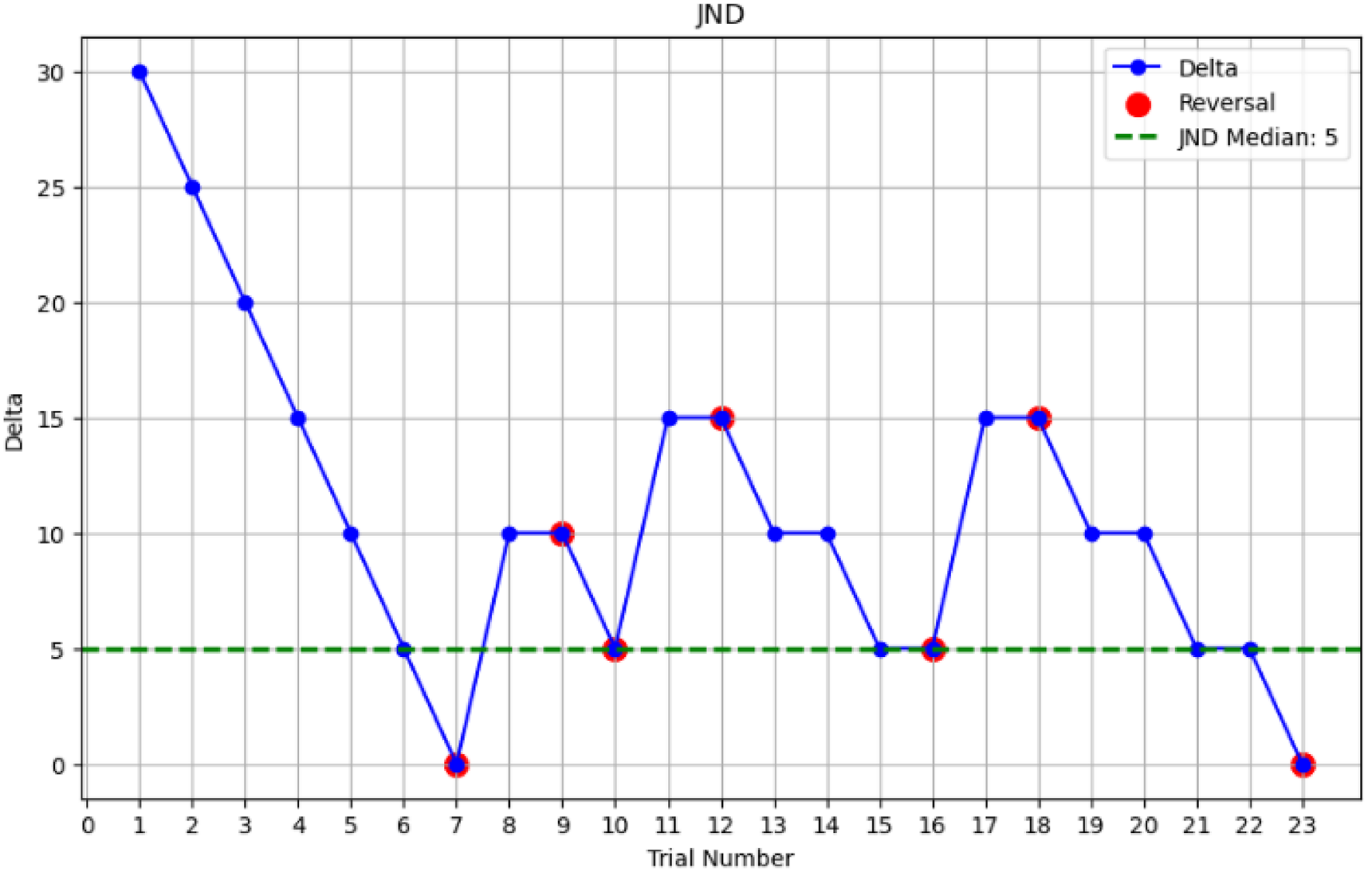
An example run of JND procedure. The maximum deformation difference between spheres, Δ, was decreased by 5% until a reversal and was increased by 10% afterward to follow the one‐down/two‐up staircase design.

### 3.7 Participants

Twenty participants (5 women, 15 men; age range: 24–33 years, μ=26.75, σ=2.29) took part in the study. An a priori power analysis conducted using G*Power (α = 0.05, power = 0.80, f = 0.25) indicated a required sample size of 21 participants. The final sample consisted of 20 participants, which closely matched this target and is comparable to sample sizes commonly used in related pseudo‐haptics and VR studies. Furthermore, 20 participants enabled a balanced Latin square counterbalancing of the five experimental conditions, resulting in four repetitions of each presentation sequence. All participants had normal or corrected‐to‐normal vision and reported no green color blindness. Participation was voluntary and no monetary compensation was provided.

Participants had varying levels of prior experience with virtual reality. Ten participants reported no previous VR experience, while the remaining participants indicated only occasional use of VR headsets, suggesting that most participants were novice VR users. Seven participants reported familiarity with the concept of pseudo‐haptics. Participants were naive to the purpose of the study and were not informed about the specific hypotheses or experimental design to minimize potential bias. Informed consent was obtained prior to participation.

The study was approved by the Ethical Committee of Friedrich‐Alexander‐Universität Erlangen–Nürnberg (Application No: 22‐275‐S) and conducted in accordance with the Declaration of Helsinki.

### 3.8 Procedure

Before the start of the user study, the participants read the information sheet and then signed the informed consent form. Then, they were introduced to the haptic glove, VR headset and controllers. After assisting participants with donning the haptic glove and VR headset and ensuring they were comfortable, the experimenters guided them through a brief familiarization stage to learn the proper way to interact with the virtual objects.

After the participants felt ready to start the study, the experiment proceeded to the JND calibration stage, where the individual stiffness discrimination threshold was determined. From this point on, the participants wore earplugs, and white noise was played through the VR headset's headphones to mask the sound of the servo motors. During this phase, participants followed instructions displayed on the screen. When the screen displayed “wait for instructions,” participants remained idle. When prompted with “Interact with spheres and choose the harder sphere,” participants used the index finger of their left hand to explore both the left and right spheres and determine which felt harder. Two buttons, labeled L and R and positioned on the left and right sides, allowed participants to register their choice with the corresponding hand. After each selection, the screen returned to “wait for instructions,” and the next set of parameters was presented in a random position. This process continued until seven reversals were reached, at which point the participant's JND was determined. The stage typically lasted 5–10 mins, depending on the participant's responses during the staircase procedure.

After a short break, the main experiment began. The order of the five feedback modalities was pseudo‐randomized using a 5×5 Latin square. Participants followed the same interaction procedure as during the JND calibration. For each modality, they guessed the stiffer sphere 20 times. At the end of each set, the scene automatically switched to the questionnaire system, where participants used the VR controller to complete the workload and presence questionnaires. After completing the questionnaires, participants were offered a break. Once all conditions were completed, participants answered two post‐experiment questions: “Had you interacted with a virtual reality (VR) environment before?” and “Were you familiar with the concept of pseudo‐haptics?” The entire experiment took approximately 90 min on average.

### 3.9 Statistical Analysis

Statistical analyses were conducted to examine the effects of different feedback modalities on the dependent measures in order to test the hypotheses regarding stiffness discrimination accuracy, experienced presence, and perceived workload. All analyses were conducted using R (version 4.5.0).

Success rate was converted to percentages. Presence questionnaire scores were centered by subtracting the neutral midpoint of the 7‐point Likert scale (3) to facilitate interpretation relative to the neutral response. NASA‐TLX scores and their subscales were centered by subtracting the midpoint of the scale (10). Overall presence and workload scores were calculated as the mean of their respective questionnaire items.

Before selecting the appropriate models, we evaluated the underlying assumptions of normality and sphericity for all dependent variables. Normality was assessed using the Shapiro–Wilk test, and sphericity was examined using Mauchly's test. Since several measures violated the sphericity assumption, we used linear mixed‐effects models (LMMs) instead of repeated‐measures ANOVA. For all the models, participant ID was included as a random intercept to capture individual differences, and the condition with deformation, color, and force feedback (DCF) was set as the baseline for comparison.

If a significant main effect of feedback modality was identified, we conducted post‐hoc pairwise comparisons using estimated marginal means (EMMEANS) with Tukey correction for multiple comparisons. This approach allowed us to evaluate pairwise differences while controlling Type I error. For correlation analyses, Pearson's product‐moment correlation was used for normally distributed variables, whereas Spearman's rank correlation was applied for non‐parametric comparisons. Statistical significance was assessed at p=0.05 for all analyses.

## 4 Results

The following subsections report the results for task success rate, presence, and workload across the different feedback modalities. Prior to the main experiment, a just noticeable difference (JND) calibration was conducted to determine participant‐specific stiffness discrimination thresholds. The calibration resulted in thresholds of 5% for eight participants and 10% for the remaining participants (μ=8%, σ=2.45%). The value of 5% corresponds to the minimum stiffness difference tested in the staircase procedure.

### 4.1 Task Success Rate

The boxplots illustrate the distribution and variability of task success rates across the five feedback modalities, highlighting differences in median performance and spread between conditions (Figure 5). Shapiro–Wilk normality test indicates that the distributions of the success rate are marginally normal (p=0.051). However, Mauchly's test indicated a violation of the sphericity assumption (p<0.001). Therefore, instead of repeated measures ANOVA, we fitted a linear mixed‐effects model (LMM) to examine the effect of feedback modality on success rate. The resulting model can be expressed as

(12) Task Success Rate∼Feedback+(1∣Participant)

where feedback modality was included as a fixed effect and participant as a random intercept to account for individual differences. The condition with all three feedback modalities (DCF) was set as the baseline for comparison.

**FIGURE 5:**
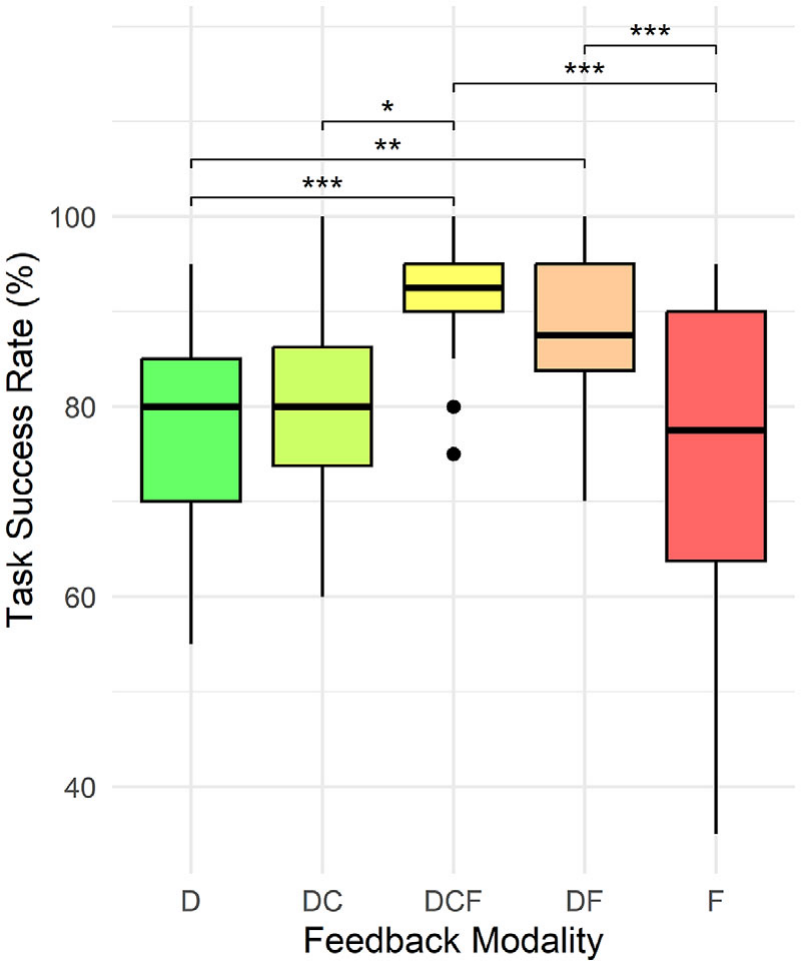
Task success rates across feedback modalities, presented within the relevant percentage range ('D' = deformation, 'C' = color, 'F' = force). Only the relevant range of the percentage scale is shown. The intersecting middle lines represent the median response, the bars show the range, and the box highlights the inter‐quartile range, with outliers indicated by •. The results suggest that pseudohaptic feedback, when combined with haptic feedback, enhances performance and can perform comparably to force feedback alone. Significance levels: *p<0.05, **p<0.01, ***p<0.001.

Type III F‐tests revealed a significant effect of feedback modality on success rate ($F_{4,76}=10.22$, p<0.001). The DCF condition yielded the highest mean success rate (μ=91%), followed by DF (μ=88.00%), DC (μ=80.50%), D (μ=76.75%), and F (μ=74.25%). Pairwise comparisons indicated that DCF significantly increased success rates compared to D (p<0.001), F (p<0.001), and DC (p=0.001), while no significant difference was observed between DCF and DF (p=0.348).

The model revealed moderate inter‐individual variability in success rates. The standard deviation of participant intercepts was 5.29, and the intraclass correlation coefficient (ICC) was 0.22, indicating that approximately 22% of the total variance is attributable to differences between participants.

Since the LMM showed a significant effect of feedback modality on success rate, we derived estimated marginal means (EMMEANS) as a post‐hoc analysis with Tukey adjustment (Table 1). In addition to the differences involving the DCF condition reported above, DF condition significantly increases success rate compared to force feedback (F) (p<0.001) and deformation feedback (D) (p=0.006) while no significant improvement is observed against DC condition (p=0.138). Moreover, differences between DC and F (p=0.293), DC and D (p=0.763), and D and F (p=0.934) are statistically not significant. Overall, the condition without any pseudo‐haptic feedback resulted in the lowest success rate (μ=74.25%). The results also suggest that a greater number of feedback channels leads to a higher success rate.

**TABLE 1:** Mean task success rate differences (Δμ) and corresponding p‐values for pairwise comparisons between feedback modalities. Differences are computed as column values minus row values. Significance levels: *p<0.05, **p<0.01, ***p<0.001.

	F	D	DC	DF
DCF	16.75% ($p<0.001^{***}$)	14.25% ($p<0.001^{***}$)	10.50% ($p=0.012^{*}$)	3.00% (p=0.879)
DF	13.75% ($p<0.001^{***}$)	11.25% ($p=0.006^{**}$)	7.50% (p=0.138)	—
DC	6.25% (p=0.293)	3.75% (p=0.763)	—	—
D	2.50% (p=0.934)	—	—	—

### 4.2 Presence

The boxplots illustrate the distribution and variability of self‐reported presence across the five feedback modalities (Figure 6). Shapiro–Wilk test indicates that the distributions of the success rate are normally distributed (p=0.121). However, Mauchly's test shows that the sphericity condition is violated similar to success rate scores (p<0.001). Therefore, we fitted a linear mixed‐effects model (LMM) to examine the effect of feedback modality on presence. The resulting model can be expressed as

(13) Presence∼Feedback+(1∣Participant)

where feedback modality was included as a fixed effect and participant as a random intercept to account for individual differences. The DCF condition was set as the baseline for comparison.

**FIGURE 6:**
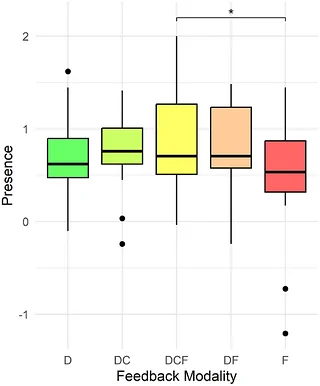
Presence ratings across feedback modalities ('D' = deformation, 'C' = color, 'F' = force). Only the relevant range is shown within the full scope of [−3,+3]. The intersecting middle lines represent the median response, the bars show the range, and the box highlights the inter‐quartile range, with outliers indicated by •. The results suggest that combining pseudohaptic feedback with haptic feedback slightly enhances presence and produces a similar level of presence as force feedback alone. Significance levels: *p<0.05, **p<0.01, ***p<0.001.

Type III F‐tests revealed a significant effect of feedback modality on presence ratings ($F_{4,76}=3.24$, p=0.017). The DCF condition yielded the highest mean presence score (μ=0.89), followed by DF (μ=0.83), DC (μ=0.76), D (μ=0.67), and F (μ=0.50).

The model revealed substantial inter‐individual variability in presence ratings. The standard deviation of participant intercepts was 0.33, and ICC was 0.43, indicating that approximately 43% of the total variance is attributable to differences between participants.

Since the LMM indicated a significant main effect, EMMEANS was calculated as the post‐hoc analysis. It validated the significant difference between DCF and F (p=0.014). Presence ratings were higher in the DF condition than in F, slightly missing statistical significance (p=0.0508). No further significant effects are found between conditions. While all conditions were rated positively above zero, the condition with no pseudo‐haptic feedback resulted in the lowest presence score, and increasing the number of feedback channels is associated with higher presence, as reflected in the mean scores.

To gain insights into the underlying factors affecting presence perception, we visualized (Figure 7) and analyzed the ratings of four sub‐factors of the Presence Questionnaire [14]: control, sensory, distraction, and realism. These factors represent different aspects of presence perception: control reflects interaction responsiveness and controllability of the environment, sensory captures the degree of sensory engagement with the virtual environment, distraction measures the extent to which external or interface‐related factors interfere with immersion, and realism reflects how consistent the virtual experience is with real‐world expectations.

**FIGURE 7:**
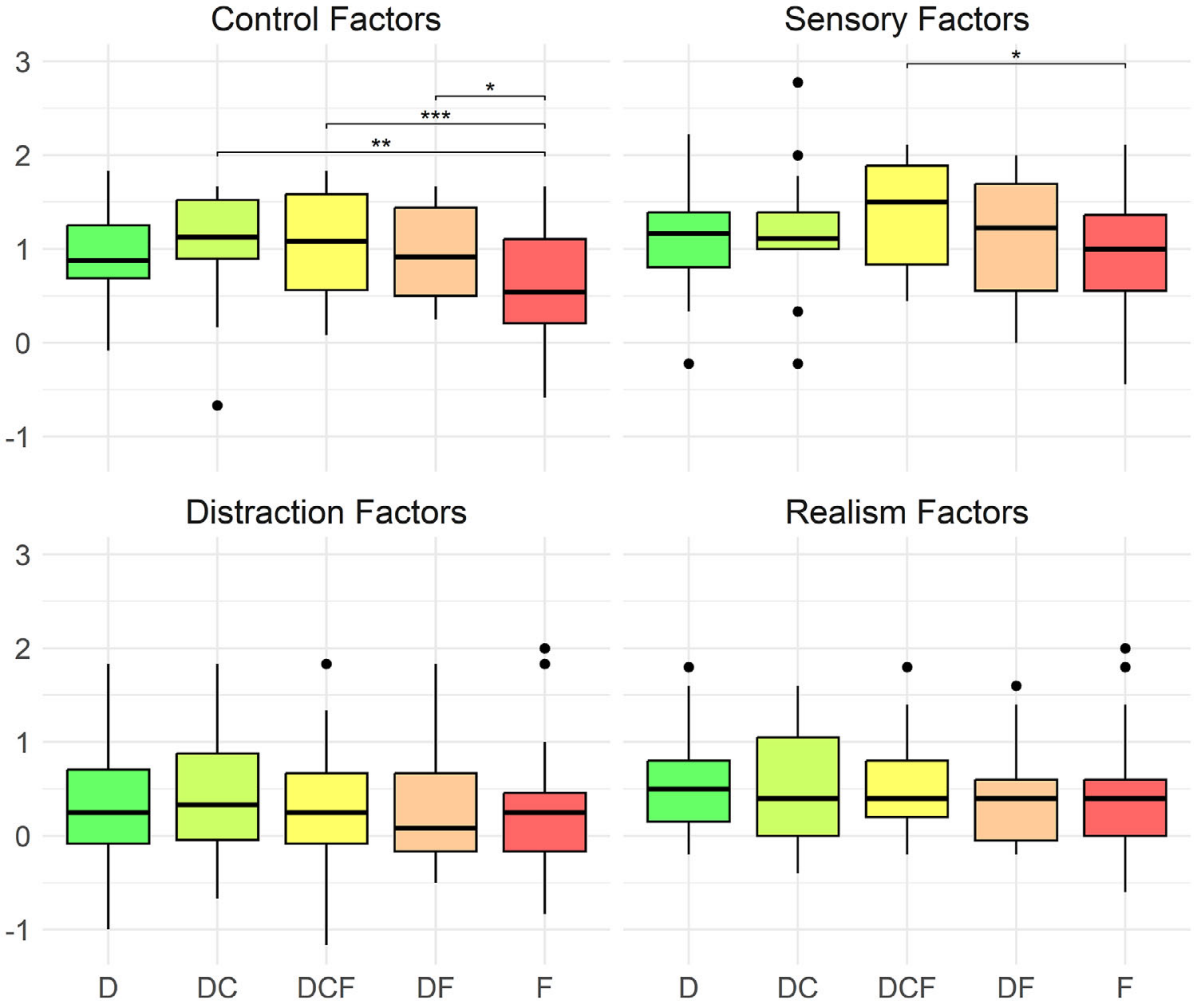
Ratings for sub‐factors of presence questionnaire [14], including control, sensory, distraction, and realism factors, across feedback modalities (‘D’ = deformation, ‘C’ = color, ‘F’ = force). Only the relevant range is shown within the full scope of [−3,+3]. The intersecting middle lines represent the median response, the bars show the range, and the box highlights the inter‐quartile range, with outliers indicated by •. A similar pattern to the presence results is observed for the control and sensory factors, while the distraction and realism factors appear unaffected by the feedback modality. Significance levels: *p<0.05, **p<0.01, ***p<0.001.

Since all sub‐factors violated the assumption of sphericity, linear mixed‐effects models (LMMs) were fitted and post‐hoc comparisons were conducted using EMMEANS when appropriate.

For the control factor, the LMM revealed a significant effect of feedback modality ($F_{4,76}=5.47$, p<0.001). The DCF condition yielded the highest mean rating (μ=1.09), followed by DC (μ=1.06), DF (μ=0.95), D (μ=0.88), and F (μ=0.58). Tukey‐adjusted post‐hoc comparisons showed that ratings were significantly higher in the DCF (p<0.001), DC (p=0.002), and DF (p=0.028) conditions compared to F.

For the sensory factor, the LMM also indicated a significant effect of feedback modality ($F_{4,76}=2.54$, p=0.046). The highest ratings were again observed for the DCF condition (μ=1.36), followed by DC (μ=1.20), DF (μ=1.14), D (μ=1.08), and F (μ=0.93). Tukey‐adjusted post‐hoc comparisons revealed a significant difference only between DCF and F (p=0.025), with no other pairwise differences reaching statistical significance.

No significant effects of feedback modality were found for the distraction factor ($F_{4,76}=0.22$, p=0.929) or the realism factor ($F_{4,76}=0.53$, p=0.716). The mean ratings for the DCF condition were relatively low for these factors (μ=0.29 and μ=0.52, respectively).

### 4.3 Workload

When interpreting workload index, the normality assumption holds (p=0.169), but the sphericity assumption was violated (p<0.001). In accordance with the boxplots in Figure 8, the LMM for workload, as shown in Equation (14), did not reveal any significant main effects ($F_{4,76}=0.74$, p=0.569). The mean task values across conditions, from highest to lowest, are: F (μ=0.48), D (μ=0.38), DCF (μ=0.14), DF (μ=−0.07), and DC (μ=−0.16) within a range of [−10,+10]. These results indicate that workload remains comparable across feedback modalities.

**FIGURE 8:**
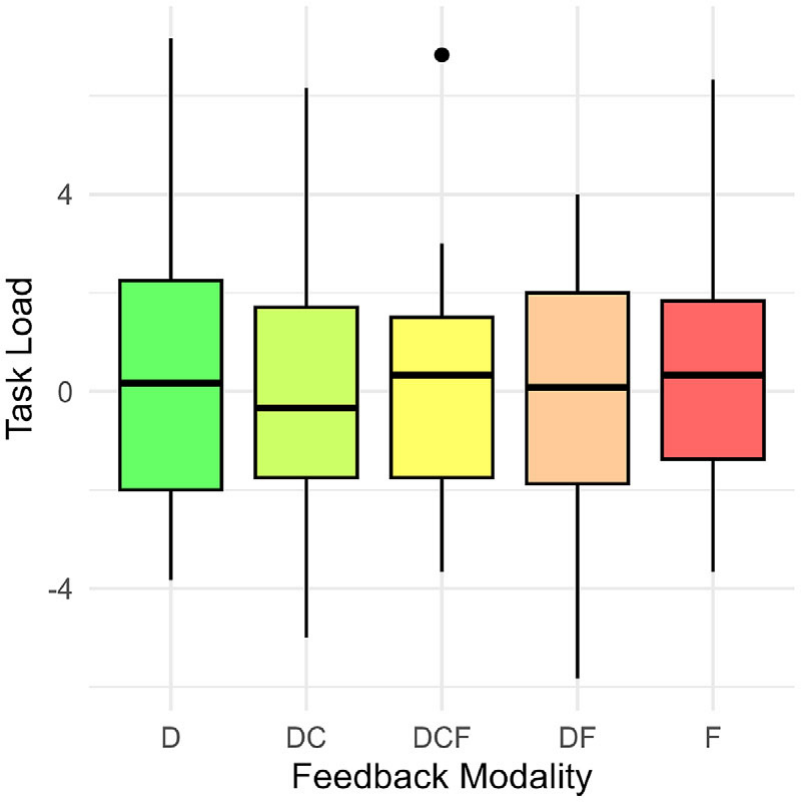
NASA‐TLX ratings across feedback modalities ('D' = deformation, 'C' = color, 'F' = force). Only the relevant range is shown within the full scope of [−10,+10]. The intersecting middle lines represent the median response, the bars show the range, and the box highlights the inter‐quartile range, with outliers indicated by •. The results indicate similar levels of workload across all conditions.

The model revealed substantial inter‐individual variability in workload scores. The standard deviation of participant intercepts was 2.34, and ICC was 0.73, indicating that approximately 73% of the total variance is attributable to differences between participants. The high ICC suggests that subjective workload is largely driven by individual differences, which may overshadow the effects of feedback modality.

(14) Workload∼Feedback+(1∣Participant)

Since some NASA‐TLX sub‐scales, such as physical and temporal demand, were expected to be less relevant to our experiment, the overall workload index may not provide meaningful insights. Therefore, we analyzed the sub‐scales individually (Figure 9). As all sub‐factors violated the assumption of sphericity, we fitted LMMs with feedback modality as a fixed effect and participant as a random intercept, using the DCF condition as the baseline. Post‐hoc comparisons were conducted using EMMEANS when necessary.

**FIGURE 9:**
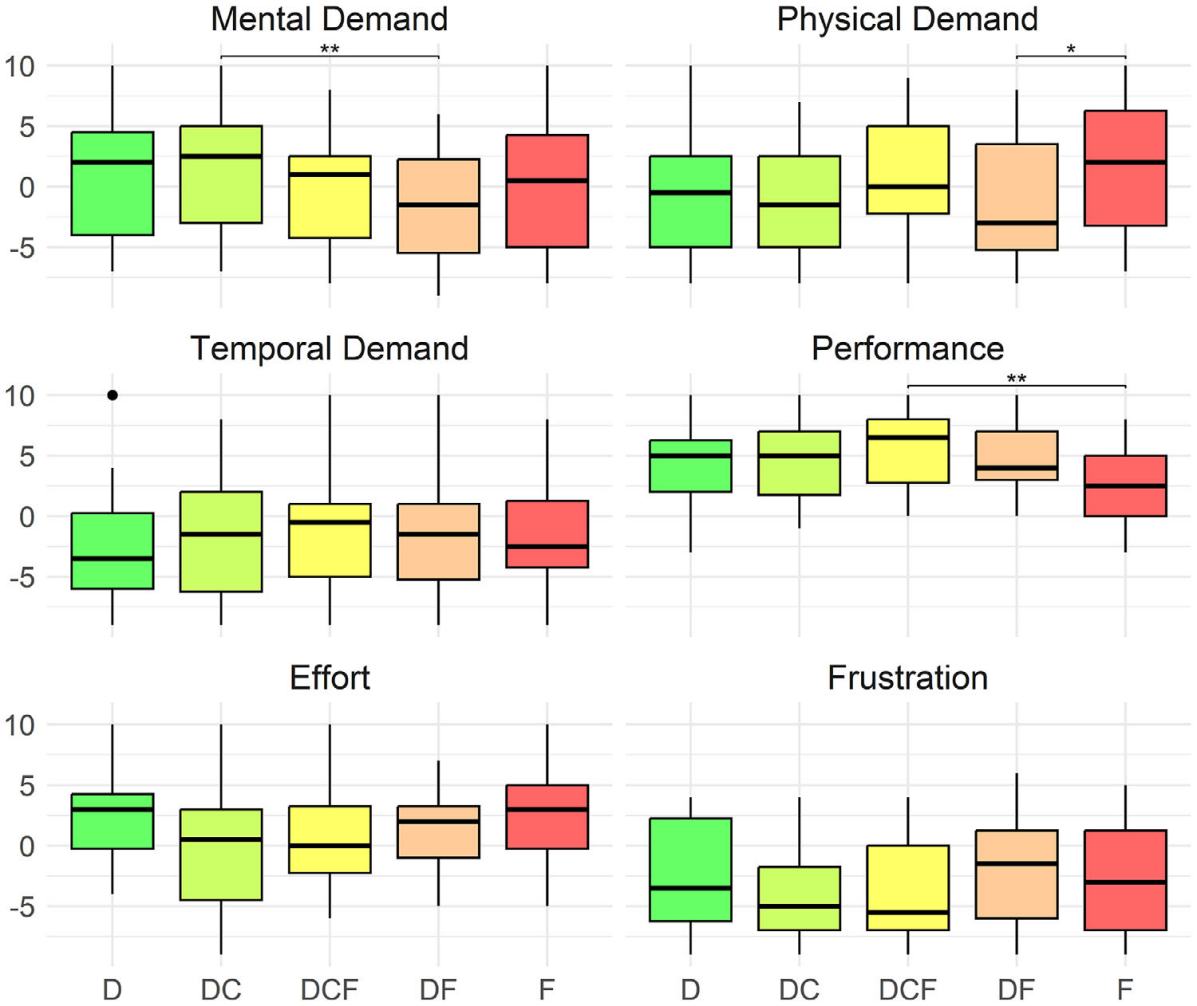
Ratings for sub‐scales of NASA TLX, namely, mental demand, physical demand, temporal demand, performance, effort and, frustration, across feedback modalities ('D' = deformation, 'C' = color, 'F' = force). Only the relevant range is shown within the full scope of [−10,+10]. The intersecting middle lines represent the median response, the bars show the range, and the box highlights the inter‐quartile range, with outliers indicated by •. Combination of haptic and pseudo‐haptic feedback appears to reduce mental demand. Performance ratings across conditions seem to align with those of success rate in Figure 5 while effort ratings show a similar pattern in an inverted manner. Significance levels: *p<0.05, **p<0.01, ***p<0.001.

Significant effects of feedback modality were observed for mental demand ($F_{4,76}=3.31$, p=0.015), physical demand ($F_{4,76}=3.17$, p=0.018), and performance ($F_{4,76}=3.71$, p=0.008). Post‐hoc comparisons with Tukey adjustment revealed that mental demand was significantly higher in the DC condition than in the DF condition (Δμ=3.15, p=0.008). Similarly, physical demand was significantly higher in the F condition than in the DF condition (Δμ=2.80, p=0.025).

For performance ratings, the DCF condition (μ=5.45) yielded significantly higher scores than the force‐only condition (F) (μ=2.70; Δμ=2.75, p=0.004). The DF condition also showed a tendency toward higher performance ratings compared to F (Δμ=2.05), although this difference did not reach statistical significance (p=0.055).

No significant effects of feedback modality were observed for temporal demand ($F_{4,76}=0.78$, p=0.540) or frustration ($F_{4,76}=1.42$, p=0.237). Effort showed a marginal overall effect ($F_{4,76}=2.41$, p=0.057), but post‐hoc comparisons did not reveal any significant pairwise differences.

### 4.4 Correlations

We used Pearson's test to examine correlations among dependent variables but found no statistically significant relationships. Additionally, we tested correlations between success rate and sub‐factors of presence and workload. Spearman's rank correlation analysis was conducted to examine the relationships between success rate and workload sub‐scales. A weak but significant positive correlation was found between success rate and performance index of NASA‐TLX (ρ=0.25, p=0.014), indicating that higher success rates are associated with slightly increased performance ratings. Conversely, a weak but significant negative correlation was observed between success rate and effort index (ρ=−0.26, p=0.009), suggesting that higher success rates correspond to lower effort ratings.

## 5 Discussion

The results of this study provide insights into both the technical design of pseudo‐haptic rendering methods and their perceptual impact during interaction. In the following discussion, we first examine the technical contributions of the proposed deformation‐based pseudo‐haptic approach and its implications for haptic interface design. We then discuss the findings of the user study and their implications for multi‐modal feedback in VR interactions.

### 5.1 Technical Contributions

Beyond the empirical findings, this work contributes a pseudo‐haptic rendering approach that leverages visually rendered deformation to augment perceived stiffness during VR interaction. By mapping interaction forces to visually observable deformation, the method introduces an additional perceptual channel that complements physical force feedback and supports accurate stiffness perception without relying solely on mechanical actuation.

A key aspect of the implementation is the coupling between deformation rendering and the interaction physics. To maintain consistent contact geometry during deformation, the interaction collider is dynamically updated as the virtual object deforms. This alignment ensures that the visually perceived deformation remains consistent with the forces generated by the physics simulation, allowing deformation cues to meaningfully contribute to perceived stiffness rather than acting as purely visual effects.

The system further enables the integration and comparison of multiple pseudo‐haptic cues, including deformation‐based and color‐based feedback. The study results indicate that deformation cues play a dominant role in improving perceptual accuracy, while color cues provide only limited additional benefit.

Together, these design choices demonstrate how lightweight algorithmic strategies can enhance perceived haptic realism and partially compensate for the limited mechanical fidelity of wearable force‐feedback devices.

### 5.2 User Study Findings

This study investigated how different combinations of pseudo‐haptic and haptic feedback influence stiffness perception, presence, and workload in VR. The results confirm that pseudo‐haptic feedback, particularly when combined with physical force feedback, can significantly enhance perceptual performance and user experience without increasing cognitive effort.

The results for task success rate support **H1.1**. Participants achieved comparable performance with pseudo‐haptic feedback (D and DC) and with force feedback alone (F). The highest accuracy was achieved under the DCF condition, which was closely followed by DF condition. Success rates improved as more pseudo‐haptic feedback modalities were combined with force feedback. These findings align with prior studies showing that multisensory integration amplifies the reliability of stiffness judgments [27, 28].

The absence of a significant difference between DCF and DF, as well as between DC and D, suggests that deformation cues, rather than color cues, primarily drive the observed performance gains when combined with force feedback. This indicates that deformation‐based pseudo‐haptic cues are particularly effective in complementing physical haptic feedback, whereas the additional contribution of color variation appears limited. One possible explanation is that color changes provide a less direct cue to material compliance than geometric deformation. While deformation visually reflects the consequences of interaction forces on the object surface, color changes require an additional cognitive association between appearance and stiffness. Consequently, when deformation information is already available, the contribution of color‐based feedback may be overshadowed by the more salient geometric cue. These findings further suggest that deformation‐based pseudo‐haptic cues can substitute for or augment physical feedback when mechanical constraints limit actuator fidelity. This observation aligns with prior work highlighting that mechanical fidelity constitutes only one component of embodied interaction, and that additional perceptual cues can support effective sensorimotor interaction in mediated environments [56]. Accordingly, **H1.2**, suggesting improved performance with increasing feedback modalities, is not fully supported. Furthermore, the lowest performance in the force‐only condition implies that visual information contributes substantially to stiffness discrimination in VR, possibly by providing more consistent spatial and temporal cues than the glove‐based feedback alone.

Similar to the task success rate, presence ratings support **H2.1**. The D and DC conditions yielded presence levels comparable to force‐only feedback, while combining pseudo‐haptic and haptic cues (DF and DCF) led to slightly higher ratings. The DF condition approached significance relative to F, and DCF achieved the highest presence overall. However, the limited contribution of color feedback suggests that **H2.2** is not fully confirmed. Among presence sub‐factors, the control and sensory dimensions appeared to benefit most from pseudo‐haptic cues, indicating enhanced agency and improved multisensory coherence [14]. These findings align with earlier results showing that pseudo‐haptics can strengthen presence through enriched visual‐proprioceptive feedback [16, 27]. Notably, realism and distraction factors remained unaffected across conditions, implying that pseudo‐haptic feedback primarily enhances agency and sensorimotor congruence rather than the perceived realism of the virtual scene. Previous studies indicate that realistic environment rendering is as important as naturalistic interaction and sensory immersion for presence [57]. Accordingly, a more realistic environment aligning with the context of interaction may further enhance perceived realism and overall presence.

Contrary to **H3**, incorporating pseudo‐haptic feedback alongside force feedback did not increase overall workload. The NASA‐TLX ratings revealed comparable workload levels across all feedback modalities. This outcome contrasts with prior work reporting increased workload due to pseudo‐haptic feedback [36, 38], but aligns with studies indicating no significant workload differences between feedback modalities [26, 33]. Sub‐scale analyses further showed that performance ratings were mildly correlated with success rate, supporting the consistency between objective and subjective measures. Overall, these findings suggest that well‐designed pseudo‐haptic feedback can complement force feedback in terms of perceptual stiffness accuracy and presence without increasing mental or physical strain, a property that is particularly desirable for long‐duration or mobile VR applications.

Collectively, the results reinforce the growing consensus that pseudo‐haptic feedback is not merely a substitute for physical actuation but a potent complement that improves perceptual fidelity through multisensory integration [27, 28]. The strong performance of deformation‐based cues underscores the importance of dynamic and spatially consistent visual information for conveying stiffness. The lack of significant differences in workload suggests that these enhancements do not come at the cost of user comfort. These results also suggest that subjective measures, particularly workload, are strongly influenced by stable individual differences, whereas objective performance is more sensitive to experimental manipulations.

However, several limitations remain. Future work should explore more realistic and contextually grounded environment rendering, ensuring that visual fidelity and scene semantics are aligned with the intended interaction to support presence and realism. In addition, more sophisticated 3D deformation rendering, such as surface shading, could further enhance compliance perception. The present study also relied on a single fingertip interaction paradigm. Extending the approach to multi‐finger or bimanual tasks may provide broader insight into how pseudo‐haptic feedback scales with task complexity and would likely require more sophisticated algorithms to handle multiple simultaneous contact points and deformation cues. Audio‐based pseudo‐haptic techniques constitute another promising approach for stiffness perception and can be implemented without additional wearable hardware. While the present study focused on visual deformation‐based cues to isolate the effects of mesh deformation, future work should investigate how auditory pseudo‐haptic feedback can complement these cues in immersive VR environments. Finally, while presence and workload were assessed using subjective measures, incorporating physiological indicators, such as pupil dilation and eye tracking, could offer complementary evidence of cognitive demand and user engagement.

## 6 Conclusion

This study demonstrates that pseudo‐haptic feedback, particularly deformation cues, can both substitute for and complement force feedback in VR, enhancing perceptual stiffness accuracy and presence experience without increasing workload. To achieve this, we introduced a deformation‐based pseudo‐haptic rendering approach that maps interaction forces to visual deformation cues, enabling perceptual augmentation of stiffness without relying solely on mechanical actuation. These findings highlight the potential of well‐designed pseudo‐haptic feedback mechanisms improving the quality of perceived force feedback when a wearable haptic interface is used or directly substituting haptic devices. By off‐loading haptic channels, these techniques could support a wider range of applications that rely on precise touch perception, such as VR training, teleoperation, and mid‐air human–computer interactions.

## Author Contributions

E.C. – conceptualization, methodology, formal analysis, software, validation, visualization, writing – original draft, writing – review & editing. X.S. – investigation, software, validation, visualization, writing – original draft, writing – review & editing. G.S. – conceptualization, resources, writing – review & editing. D.P. – conceptualization, resources, writing – review & editing. P.B. – conceptualization, methodology, resources, supervision, validation, writing – review & editing.

## Conflicts of Interest

The authors declare no conflicts of interest.

## Data Availability

The data that support the findings of this study are available from the corresponding author upon request.
